# A case study of Fe_2_TaZ (Z = Al, Ga, In) Heusler alloys: hunt for half-metallic behavior and thermoelectricity

**DOI:** 10.1039/c8ra04433c

**Published:** 2018-12-07

**Authors:** Shakeel Ahmad Khandy, Ishtihadah Islam, Dinesh C. Gupta, Muzzammil Ahmad Bhat, Shabir Ahmad, Tanveer Ahmad Dar, Seemin Rubab, Shobhna Dhiman, A. Laref

**Affiliations:** Department of Physics, Islamic University of Science and Techonology Awantipora Jammu and Kashmir 192122 India shakeelkhandy11@gmail.com; Department of Physics, Jamia Millia Islamia New Delhi 110025 India; Condensed Matter Theory Group, School of Studies in Physics, Jiwaji University Gwalior-474011 MP India; Department of Physics, National Institute of Technology Srinagar-190006 India; Department of Applied Science, Punjab Engineering College (Deemed to be University) Chandigarh India; Department of Physics, College of Science, King Saud University Riyadh Saudi Arabia

## Abstract

We have computed the electronic structure and transport properties of Fe_2_TaZ (Z = Al, Ga, In) alloys by the full-potential linearized augmented plane wave (FPLAPW) method. The magnetic conduct in accordance with the Slater–Pauling rule classifies them as non-magnetic alloys with total zero magnetic moment. The semiconducting band profile and the density of states in the post DFT treatment are used to estimate the relations among various transport parameters such as Seebeck coefficient, electrical conductivity, thermal conductivity, and figure of merit. The Seebeck coefficient variation and band profiles describe the p-type behavior of charge carriers. The electrical and thermal conductivity plots follow the semiconducting nature of bands along the Fermi level. The overall measurements show that semi-classical Boltzmann transport theory has well-behaved potential in predicting the transport properties of such functional materials, which may find the possibility of their experimental synthesis for future applications in thermoelectric technologies.

## Abstract 2

Crystal structure in conventional unit cell for Fe_2_TaZ (Z = Al, Ga, In) in *Fm3̄m* configuration.

## Graphical abstract

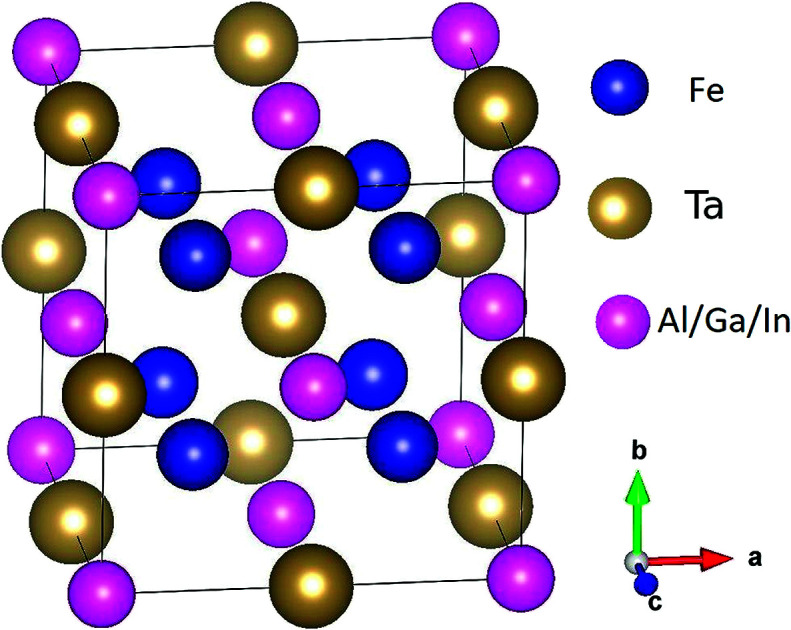

## Introduction

Ternary Fe-based Heusler structures constitute a vast family with semiconducting or half-metallic band profiles. These alloys possess excellent properties such as 100% spin polarization, topological phase transition, piezoelectric and thermoelectric power generation, and large curie temperatures.^1–4^ Alloys having stabilities w.r.t. temperature, pressure, and exposure to harmful radiations and thus regarded as potential thermoelectric materials. In thermoelectric materials, temperature gradients are expensed to produce electric current, which possibly attracted the attention of energy conservationists as well as researchers worldwide. Hence, to fix the parameters for the performance of thermoelectric materials in a simple way, the figure of merit (*zT*) has become a handy tool for researchers.1
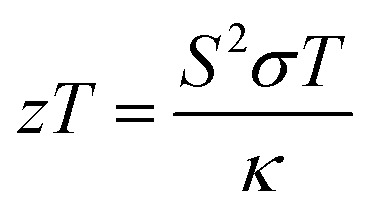
where *S* is the Seebeck coefficient, *T* is the absolute temperature, *σ* is the electrical conductivity, and *κ* is the total thermal conductivity.^5,6^ Mathematically discussing this relation, we can argue that a good thermoelectric material should have a maximum *S*^2^*σ* (called power factor), and a low thermal conductivity *κ*. It is very difficult to find a suitable material for thermoelectric application with such ideal properties (lower thermal conductivity and higher power factor) because both these quantities are strongly coupled and depend on the crystal structure and electronic properties of a material. DFT has been proved to be a powerful tool to investigate new materials for intriguing properties in a much more adequate way.^7,8^ Till date, numerous thermoelectric materials have been investigated; among them, the experimentally proven efficient material is Bi_2_Te_3_.^9–11^ For pure Bi_2_Te_3_, the highest *zT* value of 0.7 at 398 K in bulk form was obtained.^12^ Presently, Heusler systems^13–15^ are being searched for the best possible thermoelectric designs, due to their small lattice and electronic interactions, which consequently minimize the thermal conductivity. ZrCoBi-based half-Heuslers with a record-high *zT* of ∼1.42 at 973 K and a high thermoelectric conversion efficiency of ∼9% at the temperature difference of ∼500 K were reported recently.^16^ Fe_2_TiAl has also been found to have thermoelectric applications.^17^ Bilc *et al.* reported the room temperature power factors of Fe-based full Heusler alloys, which are 4 to 5 times larger than that of classical thermoelectrics.^18^ However, limited information is available on the electronic structure of Fe_2_TaZ (Z = Al, Ga, In) alloys. In addition, the untouched bonding parameters and thermoelectric properties are necessary to understand the intriguing physical properties and hence, in this study, we tried to investigate their electronic structure and thermoelectric properties in detail.

## Computational methodology

We used the density functional theory (DFT) and post DFT methods as embedded in the Wien2k simulation package^19^ to calculate the different properties of these alloys. Herein, the DFT method reflects the use of the Perdew–Burke–Ernzerhof generalized gradient approximation (PBE-GGA)^20^ and the modified Becke Johnson approach (mBJ)^21^ for exchange correlation. The appropriate cut-off parameter in the basis set was used to control the converged ground-state energy. The convergence criteria for energy and the integrated charge difference between two successive iterations were set to 10^−4^ Ry and 10^−4^ a.u^3^, respectively. The transport properties were investigated using the BoltzTrap simulation package.^22^

## Results and discussion

### Structural properties and semiconducting electronic structures

**Fig. 1:**
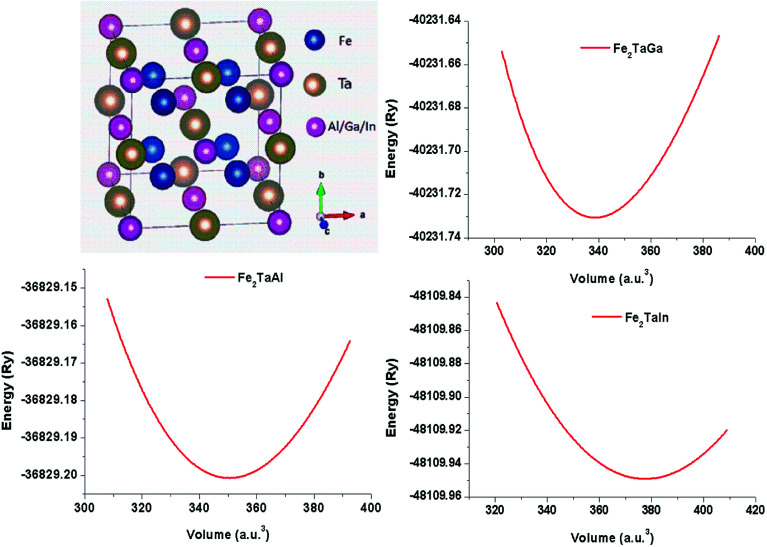
Crystal structure in conventional unit cell (left) and energy *versus* volume plots (right) for Fe_2_TaZ (Z = Al, Ga, In) in *Fm*3̄*m* configuration.

**Fig. 2:**
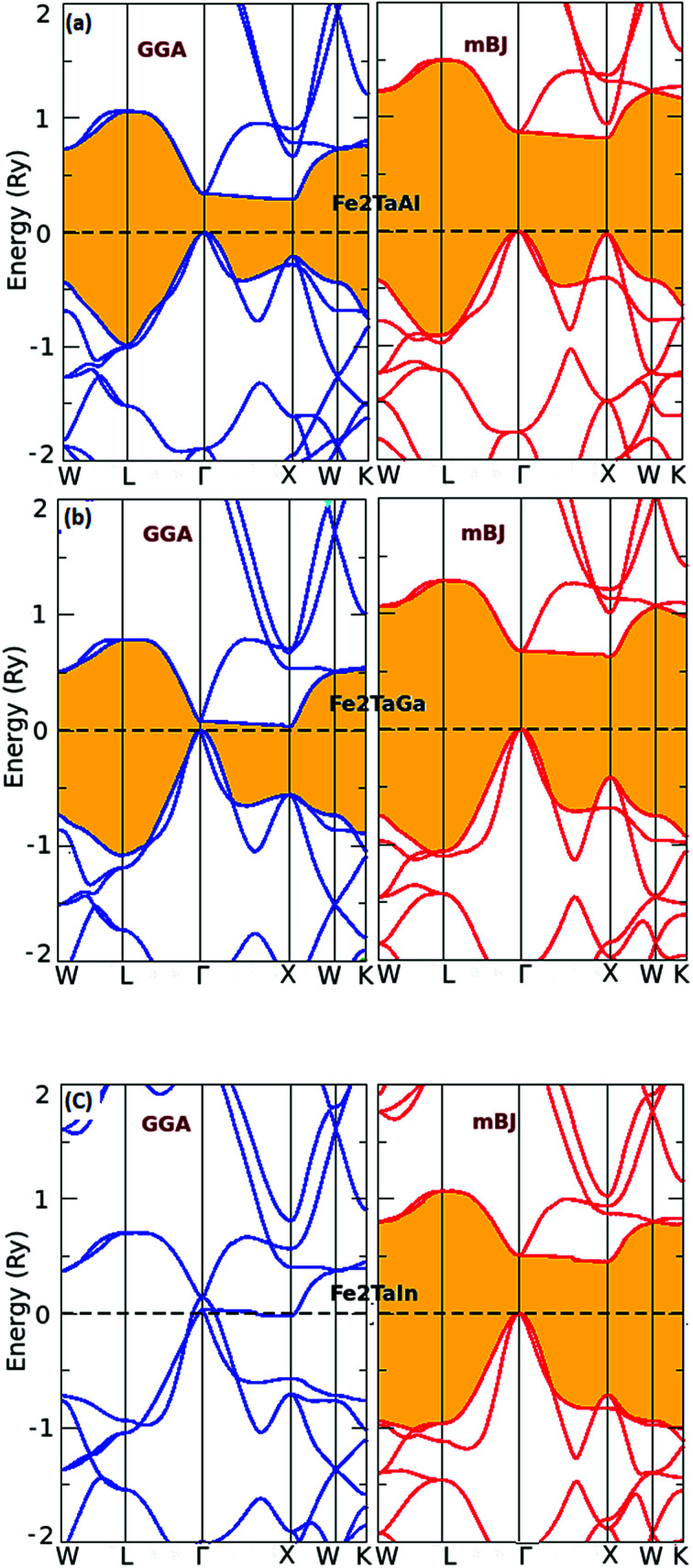
Calculated band structures of (a) Fe_2_TaAl and (b) Fe_2_TaGa (c) Fe_2_TaIn alloys by GGA (blue) and mBJ (red) methods.

The usual full-Heusler alloys of X_2_YZ stoichiometry were found to stabilize in the Cu_2_MnAl (L_21_) structure. In the present case, Fe takes the position at (1/4, 1/4, 1/4), Ta at (1/2, 1/2, 1/2) and Z at (0, 0, 0) with the space group *Fm*3̄*m*, as shown in Fig. 1.^11^ The relaxed lattice parameters obtained from energy *versus* volume plots (see Fig. 1) were used to calculate the ground-state properties, which include transport coefficients and electronic structure of the Fe_2_TaZ (Z = Al, Ga, In) alloys (Table 1). Lattice constant and consequently the volume decrease from Al to Ga indicate the screening effect of 3d states in Ga rather than in Al. However, the increase from Ga to In, refers to the large covalent radii of the In atom. Since GGA scheme has been found to underestimate the electronic structure of transition metal alloys, we employed the modified Becke Johnson (mBJ) scheme and the observed band profiles within these approximations are displayed in Fig. 2. Fe_2_TaAl and Fe_2_TaGa are observed to be semiconductors when GGA (blue) scheme is utilized, with the corresponding small energy gaps of 0.27 eV and 0.05 eV, respectively; at the same time, Fe_2_TaIn (blue) displays metallic character. However, mBJ approximation (red) clearly widens the gap between valence and conduction band in all the three compounds. Within the mBJ calculations, valence band maximum (VBM) occurs at the Γ symmetry point (at 0.00 eV) and the conduction band minimum (CBM) occurs at the X symmetry point (0.80 eV for Al, 0.61 eV for Ga and 0.45 eV for In) in their respective Brillouin zones. Thus, an indirect nature of band gap is observed in all the three materials under study.

**Table 1:** Calculated values of the lattice constant (*a*_o_), ground-state energy (*E*_0_) and energy gaps (Δ*E*) of Fe_2_TaZ alloys

Compound	*a*_o_ (Å)	*E*_0_ (Ry)	Δ*E*_GGA_ (eV)	Δ*E*_mBJ_ (eV)
Fe_2_TaAl	5.92	−36829.20	0.27	0.80
Fe_2_TaGa	5.85	−40231.73	0.05	0.61
Fe_2_TaIn	6.04	−48109.93	0.00	0.45

**Fig. 3:**
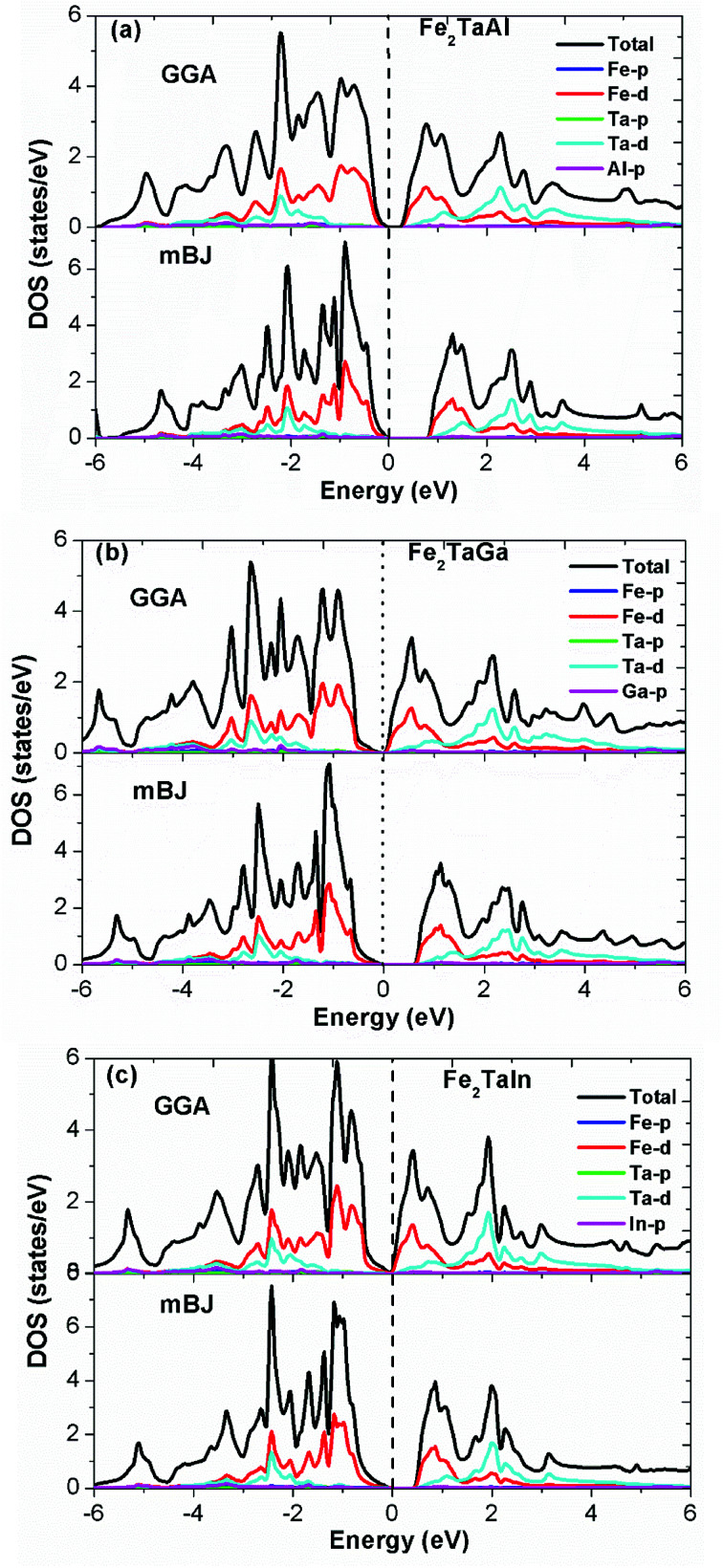
Observed total densities of states (DOS) and partial densities of states (pDOS) of (a) Fe_2_TaAl, (b) Fe_2_TaGa and (c) Fe_2_TaIn compounds calculated by mBJ scheme.

Furthermore, the calculated total (TDOS) and partial densities of states (PDOS) for the Fe_2_TaZ compounds are displayed in Fig. 3(a–c). The Fe-d and Ta-d states are mostly populated around the Fermi level with maximum contribution towards the total DOS; consequently, the corresponding bonding–antibonding states control the energy gap formation. At the same time, group IV atomic states are less active around the Fermi level in these materials. Thus, the observed band gap in these alloys is due to the typical d–d hybridization between the valence states of Fe and Ta atoms and the same has been explained elsewhere for similar materials.^2,23^ Hence, from the observed band profiles and densities of state plots, all the three compounds are found to be p-type indirect band gap semiconductors.

### Magnetism and bonding

The estimation of magnetic moment of the 3d-transition metal compounds and their derivatives was first estimated by Slater and Pauling.^24^ They related the average valence electron count (*Z*_t_) per atom to the total magnetic moment (*M*_t_) by the following equation:

2 *M*_t_ = *Z*_t_ − 18 or *M*_t_ = *Z*_t_ − 24 or *M*_t_ = *Z*_t_ − 28

Since the alloys under study have 24 valence electrons (*Z*_t_) in their equilibrium structures, the Slater–Pauling rule (*M*_t_ = *Z*_t_ − 24) comprises the zero-spin magnetic moment for all these materials. Their non-magnetic character has earlier been reported in ref. 11. Hence, from the overall analysis of electronic densities of states and magnetic studies, the present set of materials are designated as non-magnetic, p-type semiconductors with indirect band gaps.

**Fig. 4:**
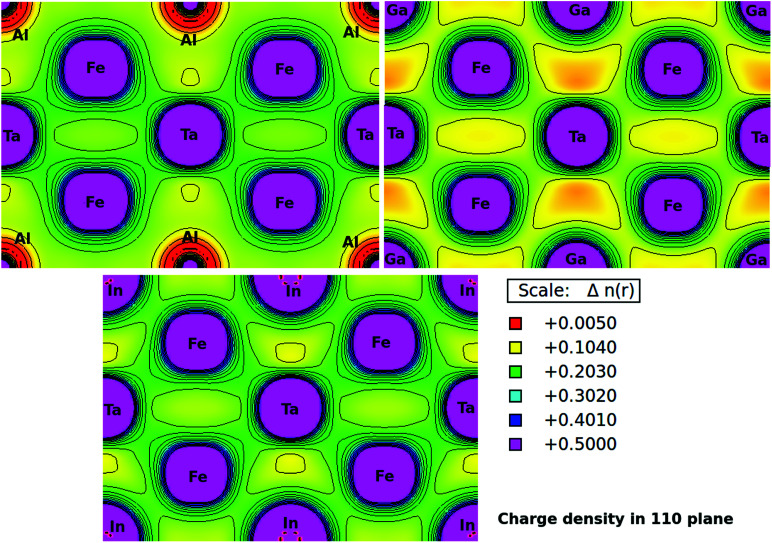
Calculated electronic charge densities of Fe_2_TaZ alloys in *Fm*3̄*m* structure in (110) plane.

Furthermore, the charge densities in the units of eÅ^−3^ for Fe_2_TaZ Heusler systems along the (110) plane were calculated and shown in Fig. 4. The electronic charge density around the constituent atoms in a lattice describes the range of interaction between them and the type of chemical bonding. From the electron density plots, it is clearly seen that the interaction between Fe and Ta and Fe and Al/Ga/In atoms in their respective compounds is covalent in nature. However, the covalent character in latter case increases as Al < Ga < In. This covalent character of bonding is also supported by the increased values of B as well as d–d hybridization preserved in these materials. The contour lines in Fig. 4 also distinguished the strength of the extent of covalent bonding in them. However, the Fe–Fe, Ta–Ta and Z–Z bonding interactions are purely ionic in nature.

### Thermoelectric properties

Under constant relaxation time approximation (CRTA), the relaxation time (*τ*), in principle, is dependent on both the band index and the *k* vector direction. However, detailed studies of the direction dependence of *τ* have shown that to a good approximation, *τ* is direction-independent.^25,26^ The same (post-DFT) method has been used to calculate the thermal transport mechanism. This method of calculation (the approach of semi-classical Boltzmann transport theory) can be understood as follows. First, the electric current of the carriers can be defined as3
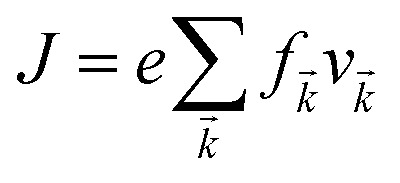
where *f*_*k⃑*_ and *v*_*k⃑*_ defines the population and group velocity w.r.t. *k⃑* quantum state, respectively, and4
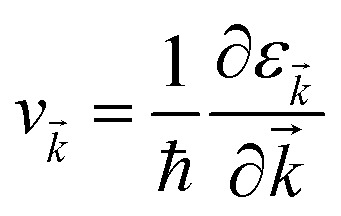


The various mechanisms accountable for the change in distribution function are linked to the electric and magnetic fields or temperature gradients, which vary the concentration due to diffusion and scattering of the carriers by phonons, impurities, *etc.* The Boltzmann transport equation is defined as5
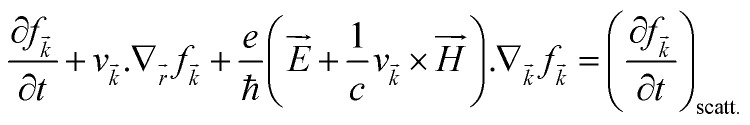


It simply gives an idea about the interaction between various intriguing parameters, and its solution defines the population of the quantum state. In the absence of external field, the Fermi distribution function *f*_0_(*ε*_*k⃑*_) gives its solution keeping the relaxation time (*τ*) constant. At the same time, the distribution function in terms of Fermi distribution function becomes6
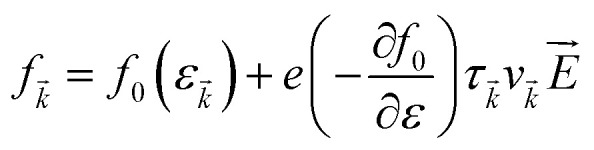
Here, 
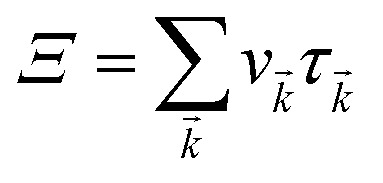
 simply defines the transport distribution function. From this function, the various transport coefficients, namely electrical conductivity, Seebeck coefficient, and thermal conductivity are calculated as follows:7
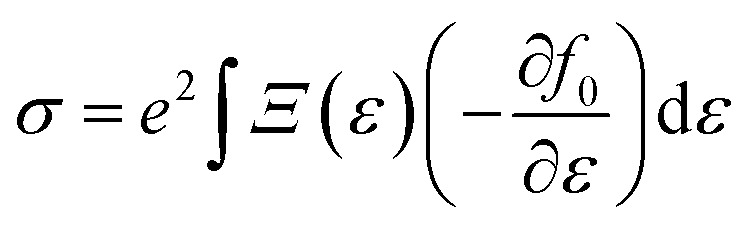
8
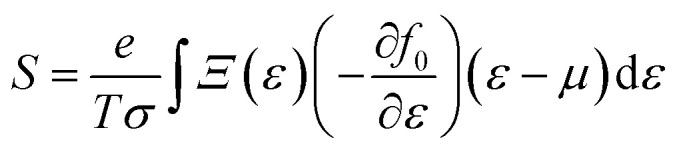
and9
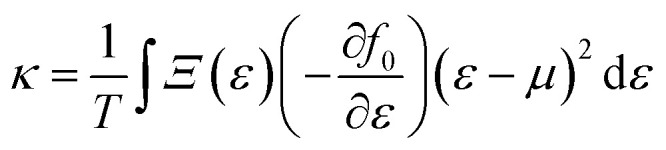


Under the assumption that the relaxation time *τ* is direction-independent, both the Seebeck and the Hall coefficients are independent of relaxation time. The above-said approximation (CRTA) positions will be appropriate if the scattering time does not vary in an energy range of *k*_B_*T*.^27,28^ Within the above-said constraints, we report the variation in the various transport coefficients such as Seebeck coefficient, electrical conductivity, and thermal conductivity with temperature.

**Fig. 5:**
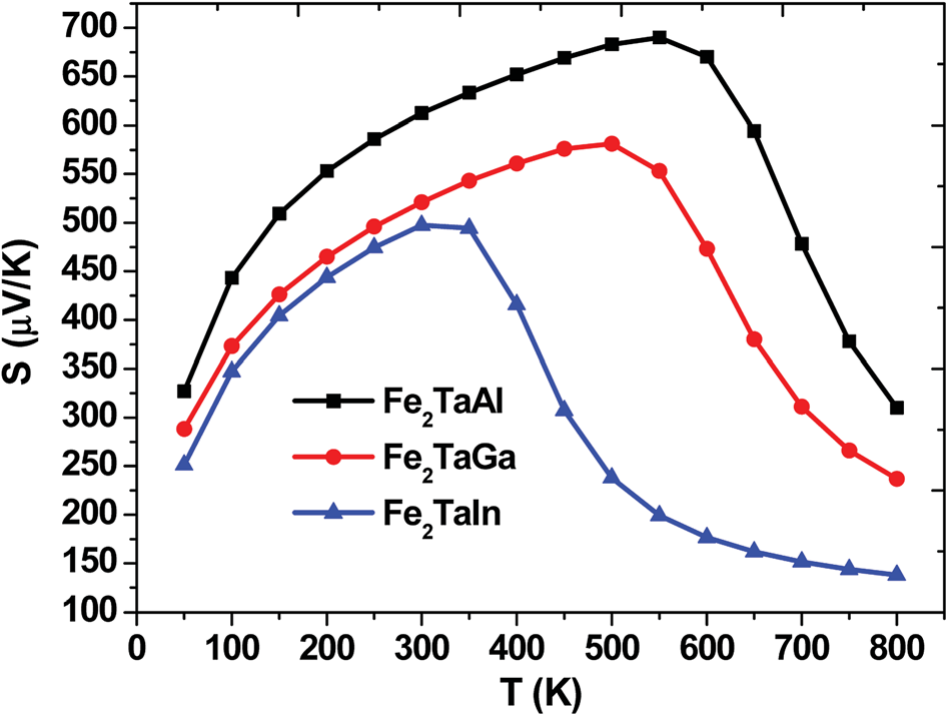
Calculated Seebeck coefficients of Fe_2_TaZ alloys in *Fm*3̄*m* structure at different temperatures for optimal doping at constant electron concentration (*n* ∼ 10^18^ cm^−3^) and relaxation time (*τ* = 5 × 10^−15^ s).

The thermopower and hence thermoelectric figure of merit (*zT*), *i.e.*, the ability to produce electric potentials w.r.t. temperature gradient, are chiefly described by the Seebeck coefficient (*S*). For the present alloys, maximum *S* is observed at 550 K (475 μK for Al), 500 K (Ga) and 300 K (475 μK for In), which thus decreases exponentially, as represented in Fig. 5. The *S* values of all these alloys are positive, suggesting the presence of holes as majority carriers in the temperature range of 0–800 K.

All these materials are p-type intrinsic semiconductors, with a dispersive conduction low band of Ta_eg_-character and Fe_eg_-band, which is flat along the Γ–X direction but highly dispersive along other directions of the Brillouin zone. This feature has been mentioned by Mahan and Sofo^29^ to be responsible for large power factors (PF). In Fe_2_TaZ full Heusler compounds (Z = Al, Ga, In), the site tends to move the Fe_eg_-dispersive band downwards on going from Al–In.

**Fig. 6:**
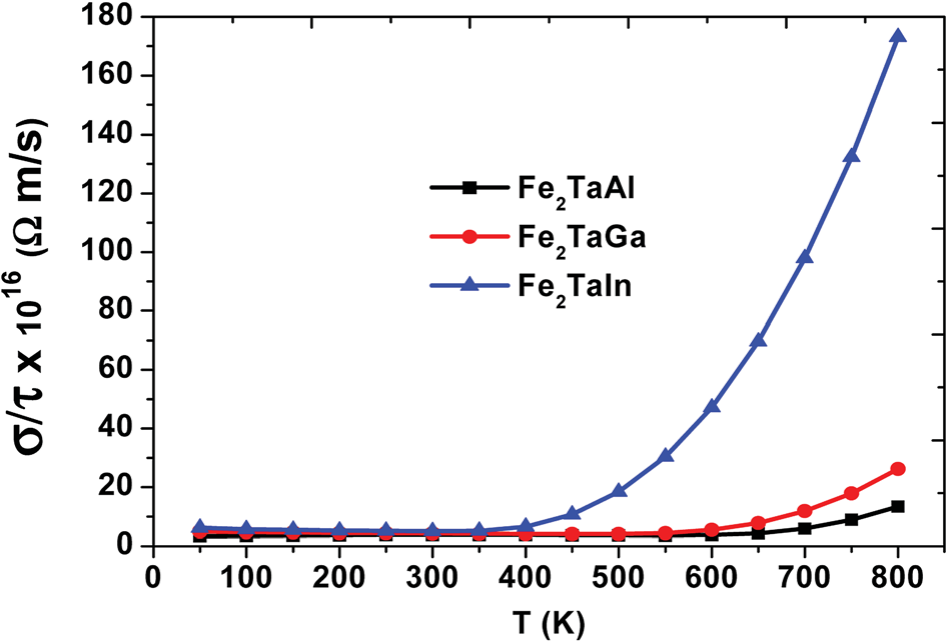
Electrical conductivity (*σ*/*τ*) as a function of temperature for Fe_2_TaZ alloys for optimal doping at constant electron concentrations (*n* ∼ 10^18^ cm^−3^) and relaxation time (*τ* = 5 × 10^−15^ s).

We have also computed the variation in electrical conductivity (*σ*/*τ*) with temperature, as depicted in Fig. 6. From 50 K to 600 K, there is almost a constant value of *σ*/*τ* (∼10 × 10^16^ Ωm s^−1^) for Al- and Ga-based alloys, which then abruptly increases, but this increase starts at 400 K for In-based alloys with much more increased values. This increase in *σ*/*τ* w.r.t temperature clearly indicates the semiconducting nature of these alloys. Among the present alloys, the maximum increase in electrical conductivity in Fe_2_TaIn is due to the lower band gap value. The semiconducting nature of these alloys allows them to show such a trend and is found suitable for the high performance of thermoelectric materials. Furthermore, the linear rise of *σ*/*τ* with temperature suggests the suitability of these alloys at both cryogenic and thermogenic temperatures.

**Fig. 7:**
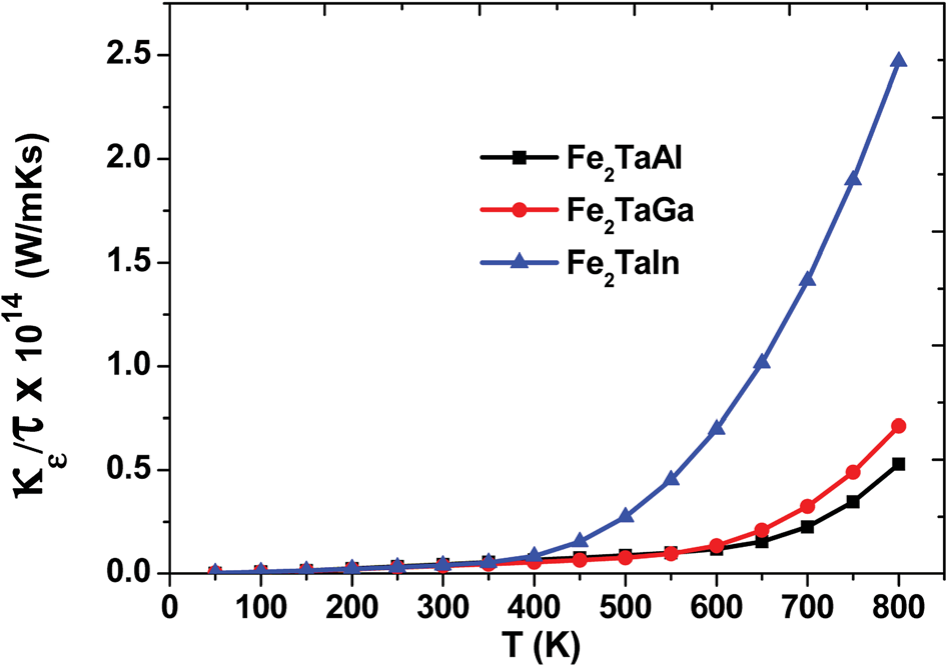
Thermal conductivity (*κ*_e_/*τ*) as a function of temperature for Fe_2_TaZ alloys for optimal doping at constant electron concentration (*n* ∼ 10^18^ cm^−3^) and relaxation time (*τ* = 5 × 10^−15^ s).

Within the constraints of CRTA, we were unable to predict the lattice part of thermal conductivity, and only electronic part (*κ*_e_/*τ*) is calculated. The variation as plotted in Fig. 7 shows an exponentially increasing trend with temperature, which clearly shows their potential use in microelectronics to dissipate the accumulated heat generated by electronic components.

**Fig. 8:**
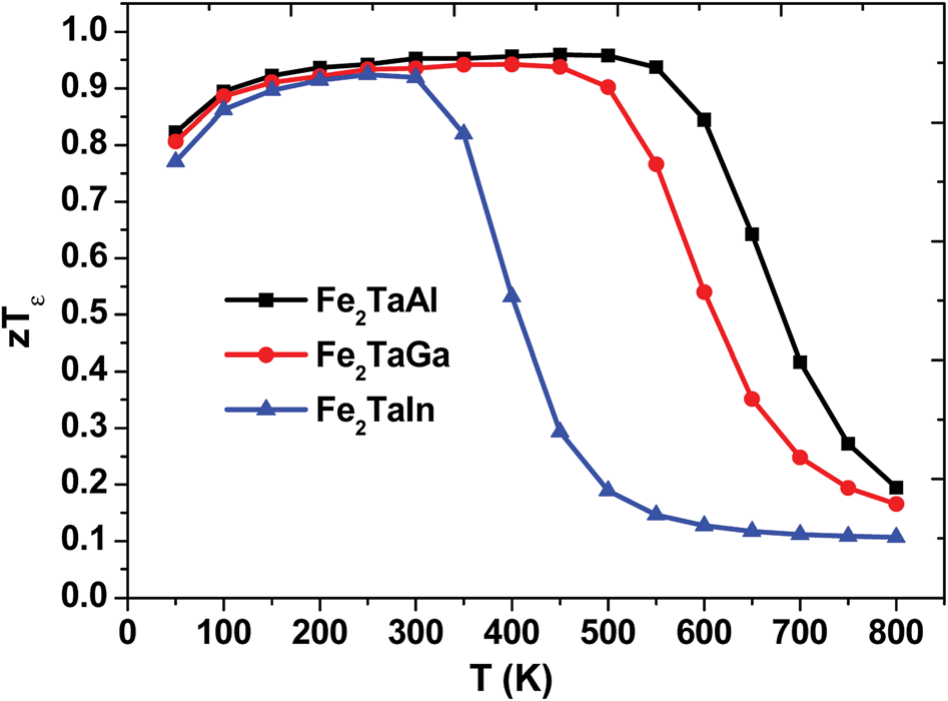
Thermoelectric figure of merit (*zT*_*ε*_) as a function of temperature.

We have also predicted the probable thermoelectric figure of merit (*zT*) and its variation with temperature, as shown in Fig. 8. Herein, we can see that the maximum *zT* for each alloy is at around 50–550 K, but Fe_2_TaIn lags behind Fe_2_TaGa, which in turn retards from the values of Fe_2_TaAl; this may be attributed to the higher values of S and lower values of *κ*_e_/*τ* in the latter case. The room temperature values of *zT* are 0.95, 0.93 and 0.91 for Al, Ga, and In, respectively. However, their maximum values reach 0.96 (at 450 K for Al), 0.94 (at 400 K for Ga), and 0.92 (at 250 K for In). This allows us to understand the maximum efficiency that a material can have with respect to the given input of heat. We have also found that replacing the lower valent s-group element with much heavier element decreases the output power. Thus, the thermoelectric efficiency of the present materials decreases with respect to temperature, and these results are found to be in good agreement with their previously reported data.^11^ Hence, the understudied work clearly recommends the stand of semi-classical Boltzmann transport theory in understanding the transport properties of these alloys.

## Conclusion

Our study describes the electronic and transport properties of cubic Fe_2_TaZ alloys from DFT calculations. Calculations of the electronic structure show the presence of indirect band gap along Γ–L symmetry for all these materials. These compounds directly fall under the Slater–Pauling rule that depicts their nonmagnetic semiconducting behavior. Thus, we have confirmed from our studies that the full-Heusler (Fe_2_TaZ) alloys are narrow band gap semiconductors with the corresponding gap of 0.80 eV for Fe_2_TaAl, 0.61 eV for Fe_2_TaGa and 0.45 eV for Fe_2_TaIn. The applicability of the semi-classical transport theory for predicting the transport mechanism of such alloys was also investigated. A reasonable agreement is seen with the previous theoretical data. The thermal transport properties convey the p-type behavior of heat carriers with *zT* depending on the band structure. The electrical and thermal conductivities follow the semiconducting behavior for all these Heusler systems.

## Conflicts of interest

There are no conflicts to declare.

## Supplementary Material
